# The relationship between diet quality indices and odds of breast cancer in women: a case–control study

**DOI:** 10.1186/s12905-023-02242-1

**Published:** 2023-03-07

**Authors:** Mohammad Hassan Sohouli, Genevieve Buckland, Cain C. T. Clark, Heitor O. Santos, Felipe L. Athayde, Vahid Sanati, Leila Janani, Akram Sadat Sajadian, Mitra Zarrati

**Affiliations:** 1 Student Research Committee, Department of Clinical Nutrition and Dietetics, Faculty of Nutrition and Food Technology, Shahid Beheshti University of Medical Sciences, Tehran, Iran; 2 Department of Nutrition, School of Public Health, Iran University of Medical Sciences, Tehran, Iran; 3 Bristol Medical School, Centre for Academic Child Health, University of Bristol, Bristol, UK; 4 Centre for Intelligent Healthcare, Coventry University, Coventry, UK; 5 School of Medicine, Federal University of Uberlandia (UFU), Uberlandia, Minas Gerais Brazil; 6 Integrative Oncology and Quality of life Department, Breast Cancer Research Center Culture, and Research (ACECR), Motamed Cancer Institute, Academic Center for Education, Culture, and Research (ACECR), Tehran, Iran

**Keywords:** Breast cancer, Diet quality, Dietary energy density, Mean adequacy ratio, Case–control

## Abstract

Dietary quality is an important factor in the etiology of breast cancer (BrCa), but further studies are required to better elucidate this relationship. Accordingly, we sought to analyze if diet quality, assessed using the Diet Quality Index-International (DQI-I), Mean Adequacy Ratio (MAR), and Dietary Energy Density (DED), was related to BrCa. In this Hospital-based case–control study, 253 patients with BrCa and 267 non BrCa controls were enrolled. Individual food consumption data from a food frequency questionnaire was used to calculate the Diet Quality Indices (DQI). Employing a case–control design, odds ratios (ORs) and 95% confidence intervals (CIs) were obtained, and a dose–response analysis investigated. After adjusting for potential confounders, those in the highest quartile of the MAR index had significantly lower odds of BrCa than those in the lowest (OR = 0.42, 95% CI 0.23–0.78; P for trend = 0.007). Although there was no association between individual quartiles of the DQI-I and BrCa, there was evidence of a significant trend across all the quartile categories (P for trend = 0.030).There was no significant association was found between DED index and the odds of BrCa in the crude and fully adjusted models. We found that higher MAR indices were associated with decreased odds of BrCa, Therefore, the dietary patterns reflected by these scores may serve as a possible guide to preventing BrCa in Iranian women.

## Introduction

Breast cancer (BrCa) is the most prevalent of all cancers, and the second leading cause of death after lung cancer [1]. Annually, more than 1.1 million new cases of BrCa are diagnosed among women, which is equivalent to 10% of all new cancers, and 23% of all cancers specifically in women [2]. Among the most important risk factors associated with BrCa include genetic risk factors, family history of cancer, smoking, sedentary lifestyle, obesity, hormone therapy, and various aspects of diet [3–5].

Extensive research has focused on the role of lifestyle-related factors, especially nutrition and diet, as preventative measures for BrCa because these factors are potentially modifiable [6]. Although studies have shown that a higher intake of saturated fatty acid and cholesterol, and a lower intake of antioxidant micronutrients, such as vitamin E, C, D, magnesium, calcium, and zinc, are associated with higher risk of BrCa [7, 8], the evidence for these specific nutrients is still inconsistent [6].

Findings of studies have shown that BrCa patients generally consume fewer vegetables, fruit, and whole grains, which are known as the main components of the DASH (Dietary Approaches to Stop Hypertension) and Mediterranean diet, than women without BrCa [9, 10]. Indeed, adherence to healthy lifestyle recommendations, including dietary guidelines, appears to be lower in those diagnosed with BrCa [11].

Research into dietary patterns, which reflect the characteristics of the whole diet rather than just specific nutrients or foods, can be advantageous because food and nutrients in the diet are generally consumed together and may therefore have interactive and synergistic effects on each other. In addition, dietary patterns are often more appropriate for extrapolation into the real-world scenario [9, 12]. Dietary Quality Indices (DQI), such as the Diet Quality Index-International (DQI-I), Mean Adequacy Ratio (MAR), and Dietary Energy Density (DED), which are indicative of the whole diet characteristics, were created to address concerns about chronic diet-related diseases [13–15]. DQI-I is a composite index at the individual diet level that was created in 2003 in order to compare the adequate amounts of diets among different cultures of countries and includes 4 components of diversity, adequacy, moderation, and balance. Another index includes DED that is defined to estimate the amount of energy in a given weight of food. MAR is also calculated based on the ratio of nutrient adequacy for energy intake and other nutrients such as vitamin A, calcium, zinc, vitamin C, riboflavin, thiamine, iron, phosphorus, magnesium, protein, potassium, and fat. It has been suggested that individuals with higher DQI-I scores may follow a healthier eating pattern, which may be attributed to a higher intake of fruits, vegetables, and dietary phytochemicals, therein reducing the risk of BrCa [16]. Previous research on diet quality indices in cancer, in particular BrCa, is fairly limited. For example, one study found no significant relationship between the DED index and the risk of BrCa [17]. Although in another study this index was associated with an increased positive risk of this disease in postmenopausal women [18], in contrast to our study, this relationship was not studied in all women. For other indexes, studies focused on other cancers, or older versions of these indexes were used to examine this association.

These DQI indices are useful tools for identifying and estimating the quality of diet in different societies with different dietary patterns around the world and their association with chronic nutrition-related diseases such as cancer, diabetes, and fatty liver disease [13, 17, 19, 20]. The relationship between diet quality and BrCa is particularly relevant to study in Iran, due to the increasing rates of BrCa as well as the unique features of the Iranian diet (for example, bulky meals, high intake of refined grains, hydrogenated fats and high percentage of energy intake from carbohydrates),the rapid nutritional transition in this region [21], and high health care costs associated with chronic diseases, especially cancer. To our knowledge, studies on investigated how these dietary quality indices (DQI-I, MAR and DED) relate to BrCa are limited. As well as other related indices that have investigated this relationship, in terms of components, they are different from our desired indices, which can cause different findings. Therefore, the present study evaluate the association between three DQI and BrCa in a hospital-based case–control study.

## Methods

This case–control study was performed on 253 BrCa patients and 267 controls, that had as of late (2019–2020) been alluded to the Hazrat Rasoul and Taleghani hospitals, Tehran, Iran. The minimum required sample size was calculated based on the ability to detect an OR of 2 with a case to control ratio of 1:1, 90% power, and a type I error ($\mathrm{\alpha }$) rate of 5% (250 participants in each group). BrCa patients were newly (< 6 months) diagnosed by an oncologist based on histopathological features of breast tumors [22]. Inclusion criteria for the case group included the following: 1) Breast cancer confirmed by an oncologist and pathology results; 2) A maximum of 6 months have passed since the diagnosis of BrCa (BrCa patients were newly); 3) Willingness to cooperate in the study; 4) Age older than 18 years and under 65 years; 5) Body mass index 18.5–40 kg/m^2^ [22]. Patients with a history of other cancers, hormone-related diseases, such as polycystic ovary syndrome (PCOS) or endrometriosis, and occurrence of metastasis as well as liquor users and long-term dietary changes were excluded from our study [22].

The benchmark or control group comprised of the people who were hospitalized for a great many non-neoplastic illnesses, with no other way of life measures, like liquor utilization, and long term dietary changes. The control group was selected from patients referred to other different departments of the clinic, like ophthalmology, muscular health, maxillofacial medical procedure, ear, nose, and throat, who were not determined to have BrCa. Also, matching people in the case and control groups based on age variables (± 5 years) and body mass index (BMI) (for three subgroups: people with normal weight BMI = 18.5–24.9, overweight = 25–29.9, with first-degree obesity = 30–34.9, and those with second-grade obesity = 35–40).

In this study, the trained dietitians were the interviewers; thus, all the participants answered all survey questions. Physical activity levels of the participants were estimated by the use of a validated short form of the International Physical Activity Questionnaire (IPAQ short form) [23]. This study was approved by the research council and ethics committee Iran University of Medical Sciences, Tehran, Iran. Written informed consent was obtained from all patients prior to participation. Also, we confirm that all methods were performed in accordance with the relevant guidelines and regulations.

### Dietary assessment

Dietary intake over the previous year was obtained using a validated semi-quantitative food frequency questionnaire (FFQ) which encompassed 168 food items [24]. The FFQ consisted of a list of usual Iranian dietary items with standard serving sizes. For each food item, the average portion size consumed, and frequency of intake were obtained via self-report on the FFQ. Frequency of intake for each food item included: never, 2–3 times/month, 1 time/week, 2–4 times/week, 5–6 times/week, and daily. Completion of the FFQ questionnaire took a maximum of 30 min. Portion sizes were changed to grams by using standard Iranian household measures [25]. Energy and nutrient intake was estimated using Nutritionist IV software. This software uses the USDA food composition table and changes have been made based on the Iranian food composition table [26, 27].

### Assessment of non-dietary exposures

In the current study, all surveys were provided by professional and trained interviewers, which led to 100% completion of the questionnaires. General information and clinical data were gathered via questionnaire, including age, age at first pregnancy (years), education and smoking status, oral contraceptive pills consumption history (months), data related to the history of breast disease (benign and breast cancer) and other cancers in self or family, bra-wearing at night, supplement and non-steroidal anti-inflammatory drugs (NSAIDs) use, and exposure to sunlight during the day. Standardized techniques were used to collect anthropometric data. A digital Seca scale (model 707, Seca, Hamburg, Germany) with a 100 g precision was used to assess body weight while subjects were unshod and wearing light indoor apparel. A tape meter was used to measure height while the subject was standing up without shoes. Weight (kg) divided by the square of height (m^2^) was used to compute BMI. A non-elastic tap was used to measure waist circumference (WC) at its narrowest point without applying pressure to the body's surface.

### Calculation of dietary quality indices

#### Diet Quality Index-International (DQI-I)

A composite indicator at the level of individual diet was created in 2003 to compare diet quality between different cultures and countries [13]. This index includes 4 parts: diversity, adequacy, moderation, and balance. The diversity part includes the general score between food groups (meat, dairy, fruits, vegetables, and grain) and the score of the diversity of protein intake among protein sources. Adequacy of this index includes adequate intake of fruits, vegetables, protein, fiber, grains, iron, calcium, and vitamin C. In the moderation section, scores related to the groups of total fat, saturated fat, cholesterol, sodium, and energy-boosting foods are considered, and finally, the last part of this index includes the total balance score between macronutrients and fatty acids. Full details on how the DQI-I score is calculated have been published previously [13].

#### Mean Adequacy Ratio (MAR)

The construction of the MAR is based on previously published methods (14). In brief, Nutrient Adequacy Ratios (NAR) were first calculated for individual nutrients. NARs for vitamin A, vitamin D, iron, zinc, calcium, magnesium, and vitamin C were calculated by age based on recommended dietary allowance (RDA) and the NAR of vitamin B1, vitamin B2, and vitamin B12 were calculated by age and based on the estimated average requirement (EAR). To calculate this ratio, the amount of each nutrient consumed was divided by the recommended standard amount. Then, the total score of these ratios was divided by the number of nutrients studied (10 nutrients) and at the end, the average ratio of nutrient adequacy was obtained for each participant [14].

#### Dietary Energy Density (DED)

To calculate diet energy density, the reported daily energy intake of each person (Kcal/d) was divided by the total weight of food consumed (g/d). The weight of drinks with no energy content was not calculated because according to previous studies, drinks are not effective in determining energy density [15].

### Statistical analyses

All statistical analyses were conducted using SPSS software (version 19.0; SPSS Inc, Chicago IL). The normality of variables was evaluated by Shapiro–Wilk tests. However, if the variables do not have a normal distribution, we use the following method. First specify Outliers and then delete them. Mean values of more than two groups were assessed using independent sample T-Test for normally distributed variables. Chi-square tests were used to compare categorical variables. The Logistic regression models were used to determine the separate association between the three different DQI’s (DQI-I, MAR and DED) and odds of BrCa, in crude and covariate adjusted models. The overall trend of OR across increasing quartiles was examined by considering the median score in each category as a continuous variable [28]. In the Tables 1, 2,3 and 4, the data were presented as mean (or N) ± standard deviation (or %) in and, statistical significance was accepted, a priori, at *P* < 0.05.

**Table 1:** Demographic, anthropometric and lifestyle characteristics of participants in case and control groups

variables	Case–Control group		*P* value^a^
	Case (*n* = 253)	Control (*n* = 267)	
	**Mean (SD)**	**Mean (SD)**	
**Age, y**	48.91(10.46)	47.13(10.08)	0.062
**BMI, kg/m**^**2**^	29.61(4.55)	29.07(5.39)	0.222
**WC (cm)**	101.15(96.39)	96.39(13.25)	** < 0.001**
**WHR**	0.96(0.08)	0.89(0.10)	** < 0.001**
**Physical Activity (Met.h/wk)**	33.18(6.11)	32.70(5.20)	0.336
**Marriage age, y**	19.43(5.02)	18.98(4.48)	0.296
**First pregnancy age, y**	22.29(5.32)	20.35(4.19)	** < 0.001**
**Child number**	2.92(1.43)	2.54(1.59)	**0.005**
	**N(%)**	**N(%)**	
**Abortion history (yes)**	94 (37.2)	78(29.2)	0.054
**Smoking current use (yes)**	8(3.2)	9(3.4)	0.894
**Tobacco current use (yes)**	8(3.2)	18(6.7)	0.063
**Employment status**			0.532
Housewife	203(80.6)	206(77.4)	
Part-time	9(3.6)	15(5.6)	
Recruitment	22(8.7)	30(11.3)	
Retired	14(5.6)	10(3.8)	
**Education status**			0.518
Illiterate	29(11.6)	24(9)	
Low education	116(46.6)	134(50.4)	
Higher education	105(42)	108(40.6)	
**Exposure to sunlight during the day (min)**			
Less than 30 min	72(28.5)	96(36)	0.215
60–30 min	82(32.4)	68(25.5)	
120–60 min	43(17)	46(17.2)	
More than 120 min	56(22.1)	57(21.3)	

A significance level of 0.05 was considered (*P*value < 0.05)

*Abbreviations*: *BMI* Body Mass Index, *WC* Waist- Circumference, *WHR* Waist-Hip Ratio

^a^Obtained from independent sample T-Test for continuous variables and Chi-square of independence for Categorical variables

**Table 2:** Medical history of participants in case and control groups

	**Groups, N (%)**		
	Case (*n* = 253)	Control (*n* = 267)	***P*** **value**^**a**^
**Family history of breast cancer (yes)**	14(5.5)	12(4.5)	0.594
**Family history of cancer (yes)**	68(26.9)	55(20.7)	0.097
**Benign breast diseases history (yes)**	20(7.9)	14(5.3)	0.224
**Menopausal status (postmenopausal)**	115(45.5)	114(42.7)	0.527
**Inflammatory disease history (yes)**	32(12.6)	35(13.2)	0.863
**Comorbidity (yes)**	93(36.8)	99(37.4)	0.888
**Night bra use (yes)**	190(75.1)	190(71.4)	0.345
**Recent special diet history (yes)**	54(21.3)	61(23)	0.647
**Vitamin D supplement (yes)**	37(14.6)	65(24.3)	**0.005**
**Herbal drug use (yes)**	68(26.9)	72(27.1)	0.961
**Iron supplement (yes)**	41(16.2)	45(16.9)	0.842
**Multivitamin mineral (yes)**	14(5.9)	19(7.9)	0.385
**Ever use of OCP (yes)**	126(49.8)	149(56)	0.156
**Anti-inflammatory drugs use (yes)**	26(10.3)	47(17.7)	**0.015**

A significance level of 0.05 was considered (*P*value < 0.05)

*Abbreviations*: *OCP* Oral Contraceptive Pill

^a^Obtained from independent sample T-Test

**Table 3:** Dietary intakes of study participants across case and control groups

	**Groups, mean (SD)**		
	Case (*n* = 253)	Control (*n* = 267)	***P*** **value**^**a**^
**Food groups (g/day)**			
Dairy	479.12 (307.21)	533.53 (334.58)	0.054
Whole grains	90.88 (87.76)	92.27(90.03)	0.858
Refined grains	336.49 (198.70)	294.60 (170.09)	**0.010**
Legumes	22.63(23.80)	33.59 (26.85)	< 0.001*
Red and processed meat	31.64 (24.73)	29.59 (20.03)	0.297
Fruits	415.98 (241.42)	513.07 (227.48)	< 0.001*
Vegetables	278.83 (154.72)	347.12 (146.46)	< 0.001*
**Nutrients**			
Energy (Kcal/d)	2753.45(798.02)	2464.1(607.43)	< 0.001*
Carbohydrate (g/d)	56.18(7.47)	54.24 (7.04)	**0.002**
Protein (g/d)	13.02 (2.15)	13.03 (2.13)	0.984
Fat (g/d)	35.11(6.75)	33.14(7.61)	**0.002**
SFA (g/d)	32.92 (11.26)	29.20(10.53)	< 0.001*
MUFA (g/d)	32.29 (13.29)	37.24 (15.97)	< 0.001*
PUFA (g/d)	20.48 (10.35)	24.49 (13.29)	< 0.001*
Cholesterol (mg/d)	293.52(135.55)	261.88(139.27)	**0.009**
Fibre(g/d)	37.96 (19.28)	39.89 (18.58)	0.247
Sodium (mg/d)	4740.74(1811.95)	4307.06(1898.50)	**0.008**
Potassium (mg/d)	3766.23(1224.29)	4297.22 (1261.12)	< 0.001*
Phosphor (mg/d)	1482.87 (492.60)	1617.48 (485.35)	**0.002**
Iron (mg/d)	20.28 (9.96)	16.34 (6.06)	< 0.001*
Calcium (mg/d)	1215.79 (463.90)	1335.27 (458.76)	**0.003**
Magnesium (mg/d)	370.06(119.89)	402.91 (133.15)	**0.003**
Zinc(mg/d)	11.76 (3.82)	12.95 (4.05)	0.001*
Vitamin C(mg/d)	159.16(89.15)	197.87 (78.89)	< 0.001*
Folate (mcg/d)	485.57 (168.28)	455.20 (163.07)	**0.037**
Vitamin B12 (mcg/d)	5.53 (3.87)	6.70 (4.53)	**0.002**
Vitamin E (mg/d)	17.64 (13.16)	23.59 (17.54)	< 0.001*
Vitamin D (mcg/d)	2.04 (3.44)	2.7 (3.06)	**0.012**

A significance level of 0.05 was considered (*P*value < 0.05)

*Abbreviations*: *SFA* Saturated Fatty Acids, *MUFA* Mono-Unsaturated Fatty Acids, *PUFA* Poly-Unsaturated Fatty Acid

^a^Obtained from independent sample T-Test

^*Statistically significant after Bonferroni correction for multiple comparisons (the threshold of statistical significance is *p*<0.0017 when presented 28 parameters are taken into account)^

**Table 4:** Mean Score of Dietary Quality Indices among Breast Cancer Patients and Control Group

	**Groups, mean (SD)**		
	Case (*n* = 253)	Control (*n* = 267)	***P*** **value**^**a**^
**DQI indices**			
**DQI-I**	51.21(10.33)	53.31 (10.95)	**0.025**
**DED**	1.23(0.20)	1.20 (0.23)	0.171
**MAR**	1.41(0.46)	1.59 (0.51)	** < 0.001**
**NAR of different nutrients**			
Zinc(mg/d)	1.47(0.47)	1.61 (0.50)	**0.001**
Iron (mg/d)	1.12(0.55)	0.90 (0.33)	** < 0.001**
Calcium (mg/d)	1.21(0.46)	1.33 (0.45)	**0.003**
Vitamin C(mg/d)	2.12(1.18)	2.63(1.05)	** < 0.001**
Vitamin D (mcg/d)	0.13(0.22)	0.18 (0.20)	**0.012**
Vitamin B2 (mg/d)	1.94(0.69)	2.25 (0.70)	** < 0.001**
Vitamin B1 (mcg/d)	1.66(0.62)	1.54 (0.55)	**0.027**
Vitamin A (mg/d)	0.98(0.69)	1.34 (0.77)	** < 0.001**
Magnesium (mg/d)	1.19(0.38)	1.29 (0.42)	**0.003**
Vitamin B12 (mcg/d)	2.30(1.61)	2.79 (1.88)	**0.002**

A significance level of 0.05 was considered (*P*value < 0.05)

*Abbreviations*: *DQI-I* Diet Quality Index-International, *DQI* Diet Quality Index, *MAR* Mean Adequacy Ratio, *DED* Dietary Energy Density, *NAR* Nutrient Adequacy Ratios

^a^Obtained from ANOVA

We classified all subjects based on the DQI-I, MAR and DED scores into quartile ranges. We adjusted the results in three models using a priori selected potential confounders, which included: model 1- age and BMI, model 2- additional adjustment for waist circumference, early gestational age, number of children, history of abortion, family history of cancer, history of inflammatory diseases, and use of anti-inflammatory drugs and vitamin D supplements, model 3- the latter model plus history of specific diet, family history of breast cancer, history of benign breast disease, and use of contraceptives. In adjusted models, confounders were used from statistical and conceptual approach respectively. In this way, the variables with *P*value < 0.2 were considered as possible confounders and were entered into the logistic regression and the odds of getting cancer was investigated. Also, in the conceptual approach of adjusting confounders in the model 3, possible confounders were selected based on clinical concepts and based on past articles and added to other confounders.

## Results

The mean ± SD for the age and BMI of the study population were 47.9 ± 10.3 years and 29.43 ± 5.51 kg/m^2^, respectively. Table 1 shows the distribution of cases and controls according to socio-demographic characteristics, smoking habits, body mass composition, and exposure to sunlight. Compared with control subjects, participants with BrCa had significantly higher waist circumference, WHR, mean age at primiparity, and number of children (*P* < 0.05). No significant differences were found for other characteristics among cases and control subjects.

Table 2 shows the medical history between the case and control groups. BrCa patients in compared with control individuals significantly had lower intake of vitamin D supplementation and anti-inflammatory drugs (*P* < 0.05). Dietary intakes of patients with BrCa (case group) and control group are shown in Table 3. Compared with control groups, individuals with BrCa had a higher intake of energy, total fats, saturated fatty acids, cholesterol, carbohydrates, sodium, folate, and iron than controls, and had a lower intake of monounsaturated fatty acids, potassium, phosphorus, calcium, and antioxidants such as zinc, magnesium and vitamins E, C and D (*P* < 0.05). Also, among food groups, dairy products, legumes, fruits, and vegetables were significantly higher in the control group than the case group (*P* < 0.05). However, refined grains were significantly higher in BrCa patients than in control subjects.

Patients with BrCa had significantly lower scores on the DQI-I (*P* = 0.025) and the MAR (*P* < 0.001). Also, in terms of components of the MAR, BrCa patients consumed less zinc, calcium, magnesium and vitamins C, A, D, B2, B1, and B12 compared to the control group (*P* < 0.05). However, DED score did not show any significant difference between the two groups (Table 4).

The odds ratio (ORs) and 95% confidence intervals (CIs) of BrCa, according to quartiles of DQI-I, MAR, and DED are presented in Table 5. In the crude and adjusted model 1 there was no evidence of decreased odds of BrCa for subjects the highest compared to the lowest quartile of the DQI-I index (OR = 0.93, 95% CI 0.52 – 1.65; P for trend = 0.074 and OR = 0.93, 95% CI 0.52 – 1.66; P for trend = 0.069, respectively). However, after adjusting for potential confounders in the model 2 and the final model, there was evidence that the odds of BrCa decreased with increasing categories of the DQI-I (p-trend 0.026 and 0.030, respectively). However, there was no evidence of an association between the individual quartiles of the DQI-I and BrCa (OR = 0.91, 95% CI 0.49 – 1.69 for model 2; and OR = 0.91, 95% CI 0.49 – 1.71 for model 3, comparing highest to lowest quartile).

**Table 5:** Odds ratio (OR) and 95% confidence interval (CI) for breast cancer based on Quartiles of DQI indices

	**Quartiles of DQI indices**					
	**Q1**	**Q2**	**Q3**	**Q4**	**OR for trend**	**P for trend**
**DQI-I**						
Case/Total (n)	69/128	66/133	63/129	55/130		
Crude model	1.00 (Ref)	1.05(0.59–1.86)	1.14(0.64–2.03)	0.93(0.52–1.65)	0.86	0.074
Model 1^a^	1.00 (Ref)	1.07(0.60–1.91)	1.12(0.62–2.00)	0.93(0.52–1.66)	0.85	0.069
Model 2 ^b^	1.00 (Ref)	0.90(0.48–1.66)	1.05(0.56–1.99)	0.91(0.49–1.69)	0.80	**0.026**
Model 3 ^c^	1.00 (Ref)	0.89(0.47–1.67)	1.05(0.55–1.99)	0.91(0.49–1.71)	0.80	**0.030**
**MAR**						
Case/Total (n)	82/130	62/130	64/130	45/130		
Crude model	1.00 (Ref)	0.66(0.38–1.14)	0.68(0.39–1.19)	0.42(0.24–0.73)	0.77	**0.005**
Model 1^a^	1.00 (Ref)	0.66(0.38–1.15)	0.73(0.41–1.28)	0.45(0.25–0.79)	0.79	**0.012**
Model 2^b^	1.00 (Ref)	0.77(0.42–1.40)	0.66(0.36–1.21)	0.44(0.24–0.80)	0.76	**0.006**
Model 3^c^	1.00 (Ref)	0.72(0.39–1.33)	0.65(0.35–1.20)	0.42(0.23–0.78)	0.76	**0.007**
**DED**						
Case/Total (n)	55/130	63/130	63/130	72/130		
Crude model	1.00 (Ref)	1.04(0.60–1.79)	1.02(0.58–1.77)	1.43(0.83–2.48)	1.11	0.230
Model 1^a^	1.00 (Ref)	0.99(0.57–1.71)	0.98(0.56–1.72)	1.44(0.83–2.52)	1.11	0.216
Model 2^b^	1.00 (Ref)	1.11(0.61–2.00)	0.87(0.48–1.59)	1.62(0.88–2.95)	1.12	0.222
Model 3^c^	1.00 (Ref)	1.08(0.59–1.97)	0.84(0.45–1.55)	1.60(0.87–2.95)	1.21	0.244

*Abbreviations*: *DQI* Diet Quality Index, *MAR* Mean Adequacy Ratio, *DED* Dietary Energy Density

^**^ Binary logistic regression was used to obtain OR and 95% CI. The overall trend of OR across increasing quartiles was examined by considering the median score in each category as a continuous variable

^**a**^Model 1: adjusted for age and BMI

^b^Model 2: waist circumference, early gestational age, number of children, history of abortion, family history of cancer, history of inflammatory diseases, and use of anti-inflammatory drugs and vitamin D supplements

^c^Model 3: adjusted for model 2 and history of recent special diet, menopausal status, family history of breast cancer, history of benign breast disease, and use of contraceptives

There was a significant reduced odds of BrCa for those subjects with the highest mean score of adequate dietary index (MAR), when compared to subjects with the lowest score, both in the crude (OR = 0.42, 95%CI 0.24 – 0.73; P for trend = 0.005) and the final adjusted (OR = 0.42, 95% CI 0.23 – 0.78; P for trend = 0.007) models, with a trend of approximately 24% reduction in the odds of cancer (OR trend = 0.76). However, no significant association was found between DED index and the odds of BrCa in the crude and fully adjusted model (OR = 1.43, 95% CI 0.83 – 2.48; P for trend = 0.230; OR = 1.60, 95%CI 0.87 – 2.95; P for trend = 0.244, respectively) (Table 5).

## Discussion

BrCa is a common disease in the population of different countries and diet is known as a potential risk factor in this disease. Extensive research has focused on the role of lifestyle-related factors, especially nutrition and diet, as preventative measures for BrCa because these factors are potentially modifiable. Also, since there is little research on diet and breast cancer and so they looked at studies of other cancers and diet to see if any similarities could be found. Through a case–control study, we investigated the relationship between DQI’s and the odds of BrCa. Overall, we identified an association between BrCa and DQI-I and the MAR index in a dose-dependent fashion; however, there was no significant difference for the DED index between groups.

More specifically, employing a test for trend, those with a higher DQI-I index had a lower odds of having BrCa in the second in and the third adjusted models. There was a 58% reduction in the odds of having BrCa for those subjects with the highest MAR when compared to subjects with the lowest score after controlling for a large number of confounders, and with evidence of a dose–response relationship. However, no significant association was found between the DED index and the odds of BrCa in the crude and final adjusted model. In one study, the range of the DED index was expressed based on the population under study [18]. This range is between 1.23 and 1.71, which is higher than the score range of this index in our study. In addition, in another study, the DQI index score range was 44 to 53, which is roughly equivalent to our score range for this index [16].

Consistent with our study, in a case–control study by Wang et al., the authors reported an inverse relationship between DQI and odds of oral and laryngeal cancer in women, whereas this relationship was not observed in men [16]. In another study, the results showed an increased risk of pancreatic cancer by following diets higher energy density, such as red meat and potatoes, and a reduction in the risk of pancreatic cancer by following diets with lower energy density, such as fruits and vegetables. In their study, there was a 72% increased risk of pancreatic cancer for subjects in the highest quintile of DED compared to the lower quintile of this index in men [29]. However, according to our findings looking at BrCa, no significant relationship was observed. Also, in a study by Vargas et al., a higher DQI-I score was associated with a reduced risk of colorectal cancer [30], whilst in a 12 year follow-up study in South Korea, the results showed that higher MAR index was associated with a reduction in cancer and cardiovascular disease mortality by 66% and 98%, in those under 30 years of age and over 30 years of age, respectively [31]. However, in Arthur et al., inconsistent to our results, the authors reported that higher intake of Western diets and higher energy density (high consumption of red meat, processed meats, refined grains, high-fat dairy and desserts), compared the Mediterranean diet with lower energy density (high consumption of fruits, vegetables, whole grains, poultry, fish and legumes), was associated with an increased odds of hormone-dependent cancers [17]. In addition, in another study of 92,225 postmenopausal women with colorectal, pancreatic, ovarian, endometrial, and laryngeal cancers, contrary to our findings, it was reported that a higher DED index was associated with an increased BMI, WC, and risk of obesity-associated—cancers [32]. Differences in study results may be due to differences in dietary patterns of different populations, study design and s sample size, different methods of measuring and estimating food intake, as well as variability in adjusted confounders. A possible explanation for the lack of association between the DED and BrCa that we observed is that in our study, unlike other studies, fiber intake increased across quartiles of DED. In a weight maintenance trial, with controlled feeding in 48 women, it was found that compared to high-fat (40% energy) and low fiber diets (12 g per day), low-fat diets (20–25% energy) with higher fiber (40 g per day) significantly reduced the serum concentration of sex hormones associated with BrCa risk (by 9 to 15%) [33]. Studies have also suggested that higher DED scores are linked to lower dietary antioxidant intake and higher insulin concentrations, which may increase the risk of cancer/tumor growth by inhibiting apoptosis, stimulating cell proliferation, and enhancing angiogenesis [29, 34].

Evidence suggests that following a healthy diet includes eating foods rich in antioxidants and phytochemicals known as anti-inflammatory compounds, as well as eating more fruits and vegetables, especially dark green vegetables can indicate a higher score of MAR and DQI-I indices that the balance between the antioxidant and oxidative systems resulting from the intake of these diets can reduce the risk of cancer by regulating cell growth and proliferation [19]. Furthermore, previous studies also suggest that higher scores of DED index, via increasing insulin concentration, can increase the synthesis of insulin-like growth factor IGF-1 and inhibit IGF-1-binding proteins, known to be a predictor of cancer and a factor associated with increasing estrogen in adipose tissue, and promoting tumor growth by inhibiting apoptosis, stimulating cell proliferation, and enhancing angiogenesis [29, 34].

The findings of our study also showed that some micronutrients, including potassium, phosphorus, calcium, zinc, magnesium and vitamins E, C and D, received less in the case group compared to the control group. Studies suggest that lower intake of these micronutrients, which are usually associated with lower fiber intake, can increase and maintain weight and body fat mass. This accumulation and storage of fat in the body is usually associated with an increase and retention of estrogen in the tissues and can increase the risk of chronic diseases, especially hormone-related cancers such as BrCa [35]. Therefore, differences in these nutrients may be clinically impactful. Special attention should be paid to vitamin D, since it plays a key physiological role in the development and function of the mammary gland [36], although the literature remains conflicting regarding vitamin D status and the risk BrCa. For instance, a meta-analysis of 9 prospective studies suggests a 12% decrease in the risk of BrCa in postmenopausal women for each 5 ng/mL increase in 25(OH) D [37]. However, in a RCT including 36,282 postmenopausal women, a reduction in BrCa (in situ) was found for those patients who underwent 400 IU/d of vitamin D3 combined with 1000 mg/d of elemental calcium carbonate [36]. In our study, the control group reported higher use of vitamin D supplements compared to the case group (24.3% vs 14.6%, *p* = 0.005). Nevertheless, due to the nature of our study design and the lack of control over the dosage across vitamin D supplements, we cannot infer that vitamin D supplements are protective for BrCa. Interestingly however, the Vitamin D and Omega-3 Trial (VITAL) represents ongoing research that may be able to elucidate the clinical magnitude of supplementing vitamin D in preventing cancer by addressing the effect of 2000 IU/d vitamin D3 with or without 1 g of omega-3 fatty acids in 25,871 healthy subjects.

One of the strengths of the current review is the powerful consideration models, which were far reaching. To the best of our knowledge this is the only study which has considered the association between DQI and breast cancer. Also, due to a maximum of 6 months having passed since the diagnosis of the disease in these patients, the likelihood of a change in their habits and eating patterns due to the disease was greatly reduced. The 168 items FFQ used in this study covers most of the foods that our study subjects received. Although this study is innovative, there are certain limitations that should be noted. Some confounders may not have been taken into account despite the fact that this study investigated all potential confounders. Despite finding evidence of a link between DQI and BrCa, the retrospective methodology of this investigation prevented us from establishing causality of the observed correlations. Therefore, this finding has to be verified in further prospective studies and RCTs. Additionally, data were gathered by self-report methods, which are known to be prone to over- or underreporting. However, we aimed to address this by utilizing skilled interviewers and instruments that had undergone thorough validation. Additionally, the statistical method was suitable for reporting at the group level. Another potential disadvantage of the research is the possibility of very modest changes between specific foods consumed during the interview and before to the diagnosis. However, the precise number of participants who altered their diet was not recorded in the research. In addition, we assessed pre-diagnosis consumption for each food item.

## Conclusion

We found that higher DQI-I and MAR indices were associated with decreased odds of BrCa. However, there was no significant association for the DED index between groups. Overall, this case–control study shows an important relationship between different scores of dietary quality indices and the risk of BrCa. The dietary patterns reflected by these scores may serve as possible guidelines for cancer prevention in pre and postmenopausal women. It seems that according to the results of the study on the potential impact of quality and content of diet including total energy intake, micronutrients and macronutrients and other risk factors such as obesity or overweight and lifestyle on the risk of BrCa, we can reducing the risk of BrCa in the community by trying to recommend and teach proper intake of a healthy and nutritious diet by relevant experts and consultants.

## Data Availability

Data is available upon request from the corresponding author for the article due to privacy / ethical restrictions.
